# BIM in *People2People* and *Things2People* Interactive Process

**DOI:** 10.3390/s20102982

**Published:** 2020-05-24

**Authors:** Bruno Mataloto, João C. Ferreira, Ricardo Resende, Rita Moura, Sílvia Luís

**Affiliations:** 1 ISTAR-IUL, Instituto Universitário de Lisboa (ISCTE-IUL), 1649-026 Lisboa, Portugal; Ricardo.Resende@iscte-iul.pt; 2 INOV INESC Inovação—Instituto de Novas Tecnologias, Lisboa, Instituto Superior Técnico, 1000-029 Lisboa, Portugal; 3 ISCTE-Instituto Universitário de Lisboa (ISCTE-IUL), 1649-026 Lisboa, Portugal; rita_moura@iscte-iul.pt; 4 CIS-IUL, Instituto Universitário de Lisboa (ISCTE-IUL), 1649-026 Lisboa, Portugal; Silvia_Luis@iscte-iul.pt; 5 Centro de Administração e Políticas Públicas (CAPP), Instituto Superior de Ciências Sociais e Políticas (ISCSP), Universidade de Lisboa, 1300-666 Lisboa, Portugal

**Keywords:** sensors, building information models, energy saving, IoT, BIM, unity

## Abstract

In this research work, we present an IoT solution to environment variables using a LoRa transmission technology to give real-time information to users in a *Things2People* process and achieve savings by promoting behavior changes in a *People2People* process. These data are stored and later processed to identify patterns and integrate with visualization tools, which allow us to develop an environmental perception while using the system. In this project, we implemented a different approach based on the development of a 3D visualization tool that presents the system collected data, warnings, and other users’ perception in an interactive 3D model of the building. This data representation introduces a new *People2People* interaction approach to achieve savings in shared spaces like public buildings by combining sensor data with the users’ individual and collective perception. This approach was validated at the ISCTE-IUL University Campus, where this 3D IoT data representation was presented in mobile devices, and from this, influenced user behavior toward meeting campus sustainability goals.

## 1. Introduction

Through IoT implementations, available data are growing exponentially, and visualization approaches are needed for fast data interpretation, especially by non-technical users whose behavior must change. IoT data representation has been recognized as one of the most significant factors impacting people energy conservation [1]. Our research results of IoT color data representation, suggesting that this approach can be used to influence user behavior toward a more sustainable behavior in shared spaces as a human dimension plays an important role in energy efficiency. Color representation plays an important role, and several studies have shown that color-coded information visualization increases comprehension [2,3,4].

The building information model (BIM) is a tool and methodology used mainly by architects and civil/structural engineers during the design and construction process. During this phase, it is used as a model of what is to be built. If the model is updated during the construction, handover, and life of the building, it becomes a digital twin, replicating the de facto contents and behavior of the installation.

The BIM is a smart model-based process that provides simulation, a virtual environment, and digital modeling. The BIM also has great potential to be used as an information representation tool for users to have a better perception of environment reality and allows user interaction that is important for energy efficiency in buildings, since users are active members of this energy management process in a building.

ISCTE-IUL’s facility management office has been developing a BIM model that is being used to feed maps, room listings, staff location, and space characteristics as well as equipment location [5,6]. This proves a significant advantage because BIM models are based on a geometric model and linked database that can be queried and updated, although currently not in real-time, with information from the university’s academic and staff management information systems.

The BIM methodology depends on standard, interoperable formats, but also on controlled information sharing that breaks down information silos, and to this end, custom-made solutions must be designed.

**Figure 1:**
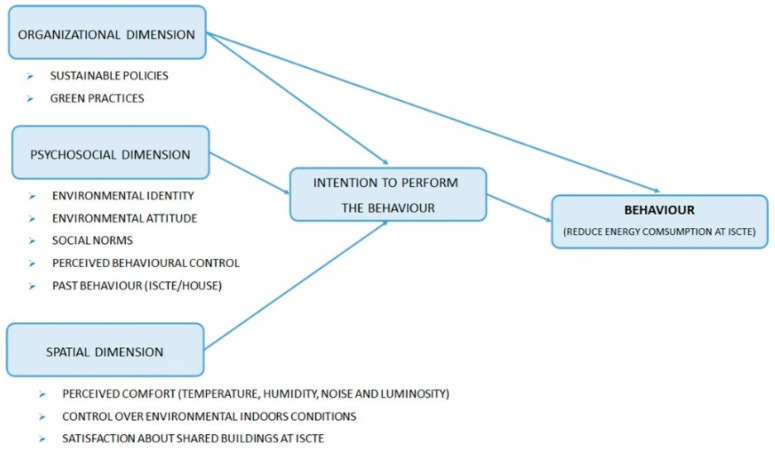
Behavior change diagnostic model for ISCTE’s community assessment.

Our research aims to answer the following question: is it possible to change individual behavior in public buildings through intuitive environmental feedback that informs on the physical context and the preferences and behavior of peers? To this aim, the developed system was oriented toward interactive processes with local users by using their mobile devices and accurate and easy to read representations of the spaces with context environment data representation as well as people’s perceptions about local temperature and light. This was performed by a multi-disciplinary team (computer science, architecture, and human behavior experts) to achieve user behavior changes in public places. The human factor plays an essential role in building energy consumption, and savings are not achieved solely through technological approaches. We performed a questionnaire Figure 1, during the first three months of 2020, at ISCTE with the participation of 622 persons (16.2% teachers and research, 74.4% students, and 9.4% staff).

Major findings at different levels were:

- Organizational: Most users stated that ISCTE has a sustainability policy, but on average, only seemed to slightly agree that ISCTE strives to implement green practices in its shared spaces.
- Psychosocial: On average, the community had a positive attitude toward sustainability and believe that they have enough capacity and autonomy to adopt more sustainable behaviors at the campus. Users also presented high levels of intention to be environmentally conscious, and some of them already performed actions to reduce energy consumption (for example, turning off lights) at ISCTE. However, users only slightly agreed that their peers engaged in these behaviors.
- Spatial: Users thought that, on average, they were neither comfortable nor uncomfortable at ISCTE, and overall, they felt slightly satisfied with its buildings.

Results showed that ISCTE and its community have a great potential to successfully engage in behavioral change interventions in the future to motivate the reduction of energy consumption, in accordance to our research goal of changing user behavior, towards building energy consumption reduction.

## 2. Related Work

### 2.1. IoT Architecture System

An IoT architecture should take into account the factors of scalability, interoperability, and reliability due to the IoT system being prepared to increase or decrease the amount of capacity needed to exchange information between the devices. An IoT architecture in the literature is usually divided into three to five layers [7,8,9,10]: (1) the perception layer; (2) the network layer; and (3) the IoT platform layer that includes the middleware or data processing layer, application layer, and some authors have also suggested the business layer [11]:

**Perception Layer**: This is the layer that involves physical objects and sensor devices that are used to collect data. Examples of this sensor data could be the location, temperature, humidity, motion, vibration, etc. This information is then passed to the network layer to have a secure transmission up to the top layers. The perception layer senses the gathered information and submits the collected data to the higher layers for intelligent processing via the IoT network layer.

**Network Layer**: This layer transfers the information from sensor devices to the processing system in a secure way. A diversity of communication protocols can be applied [12], but new ones have been developed to be used by sensors powered by a battery such as the low power wide area network (LPWAN) [13]. Examples of this are the LoRa [14] and NB-IoT [15]

**Middleware Layer**: This layer is responsible for information processing and makes decisions based on the information exchanged by the IoT system [16]. Each IoT device connects and communicates with only the IoT devices, which use the same type of service.

**Application Layer:** This layer may provide global management of the application based on the information processed by the IoT devices in the bottom layer (middleware layer). According to the ITU-T Y.2060 recommendation, the application layer contains the authorization, authentication, application data confidentiality, integrity and privacy protection, and the security audit [17]. The application layer provides services as per user demand and the processed information that comes from the lower layers is used to produce useful services for the customers [18].

**Business Layer**: This layer is responsible for the management of the overall IoT system including applications and services [19].

### 2.2. Building Information Model (BIM) as a Digital Twin

The concept of the digital twin, a virtual model that reproduces the characteristics of an existing physical asset is not new. NASA maintained a working (physical) reproduction of the Apollo capsule on Earth to allow engineers to safely test solutions to problems during missions. A digital twin [20] is an up-to-date and accurate copy of the physical asset that reproduces its characteristics and responses. It can be used to understand the behavior, test hypotheses, and predict future performance.

A building information model (BIM) is both a methodology for the management of building and construction activities that has a strong emphasis on collaboration between stakeholders and a 3D detailed geometric description of a facility and its contents: mechanical systems, furniture, and equipment. In a BIM, information and geometry are associated, for example, a door is represented by a 3D geometry, which can be more or less faithful to reality, and by the information about materials, intrusion, and fire-resistance rating or lock type, or any other relevant data. Abstract entities such as spaces are also present in BIMs as well as information about the activities or occupants.

The BIM implementation in the design and construction phases of buildings and facilities is moving at a great pace [21]. Adoption in the final stages of building life-cycle, namely facilities operation and decommissioning, is weaker, but there are already significant BIMs in facility management [22,23].

Autodesk’s Revit [24] is one of the most widely used BIM software, and, like most of them, it is built over a database that can be accessed through the software’s GUI or an API [25]. NET compatible languages such as VB.NET and C# renders it possible to connect the model to external information systems and import and export building-related information. The Dynamo visual scripting [26] language gives access to Revit’s API through visual programming. In the case of relatively simple applications that do not demand high performance, Dynamo allows for quicker development, although the code’s maintenance cost is higher than using formal programming languages (Python or C#) and Revit’s API.

Revit is a support tool for model development in the design and construction phase, and because its features are focused on that scope, this tool is not well suited to aggregate data from exterior sources or to display information to the public, lay users, or operators. Therefore, what is needed is a platform that holds the building’s geometric and non-geometric description including equipment and sensors, spaces, and activities, where this information is static, or at least updated infrequently, and receives information from sensors, connect it to the spaces, equipment, and activities and displays it to users in accessible ways, providing context and meaning to that information. We have, then, three sources of information: the BIM describes the building; the campus management platforms describe activities; and occupiers and sensors describe the environment. All three are related and interdependent and were visualized in a custom-built Unity platform.

### 2.3. BIM and IoT Integration

In [27,28,29], the authors deployed environmental and energy sensors in multiple schools to engage with students by using a gamification platform called “GAIA”, where teachers show students ways to reduce energy consumption, and the platform gives real-time feedback. One of the first problems the authors had was the data availability from the schools due to network problems that caused daily data interruptions. The technology we used was low-power oriented and long-range, giving us the advantage of placing sensors in the best location possible without changing the battery for several months. The gamification platform gives users the possibility of understanding what happens in real-time, however, numeric data are not as relevant as visual content such as a 3D view of a user current location, and many types of users do not understand technical analysis and variables. An augmented reality app and IoT development kits were used to increase the students’ desire to interact with the system. Large universities and other small schools may have many students with very low electronics knowledge or interest, which can reduce social cooperation over time.

The authors in [30] used a primary school’s BIM model to perform an energy study of the building. By using IoT devices and weather services, the authors correlated indoor and outdoor air temperatures with the weather forecast to estimate temperature trends. Despite being mentioned, energy consumptions or occupancy data were not integrated into this solution. Unlike our approach, users cannot interact with the system, since there is no user interface such as a web or mobile application to present real-time data. BIMs are commonly used as a ground tool to perform building analysis, however, in our research work, we used them as a real-time digital-twin.

Another research work [31] suggested a risk management system in a hospital context. The authors presented a BIM framework to merge information from CCTV cameras and fire detectors. Despite the high importance from a public safety viewpoint, the proposed technology is very sensitive to Internet or power outages, compromising the system’s effectiveness.

IoT systems integrated with BIMs are also being used to provide indoor localization data.

In [32], Bluetooth low energy (BLE) sensors were attached to construction workers’ hats to collect localization and environmental data. BIMs were used to choose optimal locations to place BLE beacons to track the workers’ positions. In our system, the BIM is part of the integrated solution and not only a tool to develop the system. In [32], environment data were not accurate due to the BLE beacons’ possible detection range and the workers’ movements.

Another campus research work [33] used a digital twin concept to integrate IoT sensors and collect real-time environment data. Their proposed system used WiFi modules that always require a nearby Internet router and a continuous power supply, unlike our system’s IoT oriented low-power and long-range technology.

AI and machine learning can also be integrated with BIM in IoT systems to estimate predictions, evaluate situations based on sensors data, or as an automated decision-making mechanism. In [34], low power sensors were deployed at a manufacturing facility to collect and present environmental data in real-time, together with a BIM of the building. In this case, the data were not merged with the model, but presented in the same software interface.

Our research work differentiates from others due to our *Things to People* approach, where users interact directly with the system through a browser or mobile application, and temperature, humidity, or light data are embedded within the 3D model using appealing, easy to understand colors and warnings.

Our communication technology is another key aspect of this work since low-power and long-range networks are still a relatively new and powerful tool for IoT applications.

## 3. Methodology and Objectives

As we wanted to develop a digital artifact, this work methodology was built upon a research-based design process oriented to the development of prototypes and that emphasizes creative solutions, the exploration of several ideas and design concepts as well as continuous testing and redesign of the proposed system.

Following recent developments on BIM modulation and IoT technologies, this research aimed to develop a low-cost and low-power IoT monitoring system to measure the real-time temperature, humidity, and luminosity of each room using LoRa technology. LoRa is an IoT oriented communication protocol with a long-range and low power consumption, which is ideal for sensors.

By using Dynamo scripting language, other campus IT services such as the room occupation system and staff and maintenance schedules will provide information to the system to be processed and presented using a detailed previously developed BIM of the campus.

The Unity 3D game engine integrated the BIM with the IoT system and represented data in real-time in the model with colors, avatars, and warnings. This approach allows users to easily perceive the surrounding environment and what can be done to reduce costs or increase comfort as well as attend to anomalies detected and displayed in the application.

Our research work intends to create social interaction with users by using low-cost, power-efficient technologies, and richly colored models as the user interface toward global sustainability goals and general efficiency.

Unity [35] is a game engine and a 2D and 3D development software that allowed us to integrate a converted BIM of a building with our system variables by introducing C# scripts into the model, and change its color according to the variable being analyzed, as described in the following sections.

**Figure 2:**
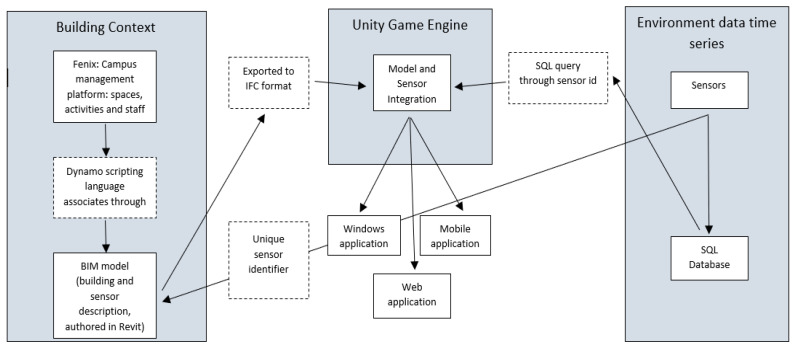
Scheme of the BIM, campus activities, and sensor data in Unity.

Figure 2 describes the integration scheme of the BIM, campus space, and staff description and sensor data.

Existing building systems such as the campus management platform where spaces, activities, staff, or maintenance events are listed provide all of the information to the BIM using Dynamo scripting language. In contrast, our IoT LoRa system provides real-time environmental data collected by the deployed sensors. The same information is also stored on a SQL database for historical process and analysis. The Unity game engine acts as an active information aggregator by querying the database and presenting the 3D BIM building together with all of the processed data. Unity allows for the application deployment on various platforms such as Windows, mobile, or web application.

## 4. IoT System

### 4.1. Perception Layer

**Figure 3:**
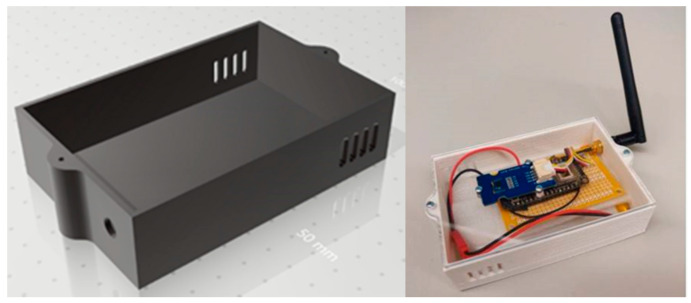
Box drawn with AutoCAD and printed in lactic polyacid (PLA) at the ISCTE-IUL FabLab.

**Figure 4:**
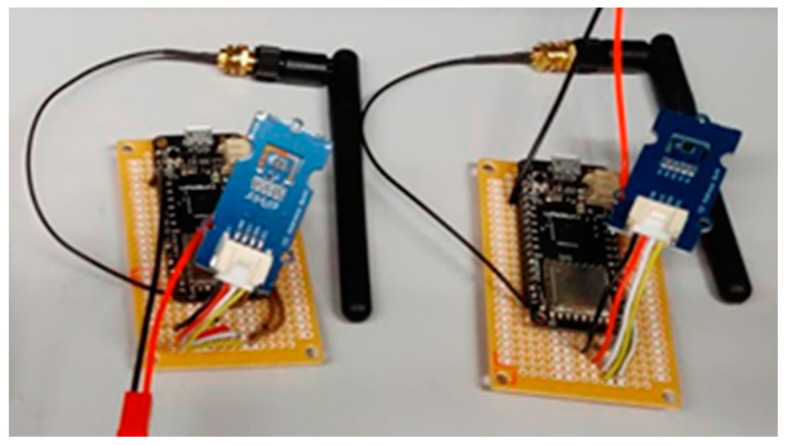
Prototypes of LoRa nodes for the data centre in the process of completion.

For prototypes intended to study classroom conditions, LoRa Seeeduino boards (LoraWan) were used together with a DHT22 temperature and humidity sensor with an accuracy of ±0.5 °C and ±2%, respectively, light sensor, and presence sensor. For the prototypes, LoRa32u4 II cards based on theAtmega32u4 microcontroller were used. These prototypes were welded to punched circuit boards Figure 3, and the entire circuit was placed inside a box drawn in AutoCAD and printed in 3D Figure 4. As these sensors are designed to be portable and easy to place anywhere, they are powered by 2400 mAh lithium batteries, the durability of which can be extended by code optimization and sleep between transmissions.

These sensor calibration processes were accomplished in our IoT laboratory: DHT22 sensors were calibrated using a high precision infrared thermometer and by comparing the real values with the sensor’s data each hour for one week; the relative humidity was also calibrated using the calculated functions presented in [36]; and motion sensors were calibrated by applying a detection limit considered as movement, thus avoiding false positives.

**Figure 5:**
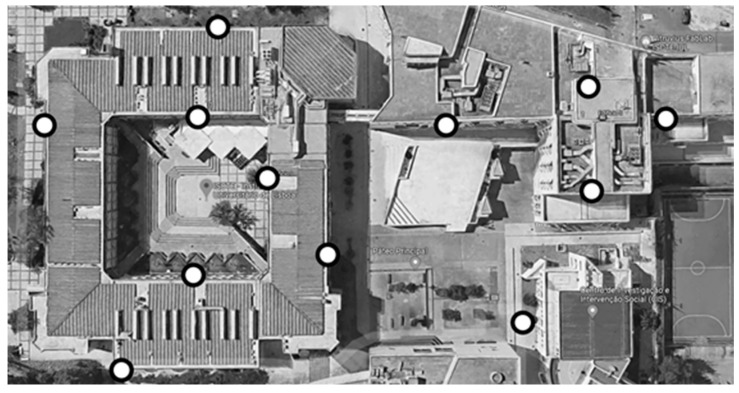
Placement of external temperature and humidity sensors.

Temperature and humidity monitoring on the exterior of the buildings Figure 5 were carried out using Lansitec sensors with an IP 65 rating (water-resistant) with built-in batteries capable of powering the sensor for five years at the transmission frequency of five minutes.

The placement of these sensors was determined with the help of ISCTE central services, based on the indications of the Sustainability Department and the Architecture and Urbanism Department. The final placement layout is visible in the following map:

The white circles mark the most interesting points of study, namely the four wings of Building I, its patio, the central atrium, and the roof area of Building II above the campus main data center.

**Figure 6:**
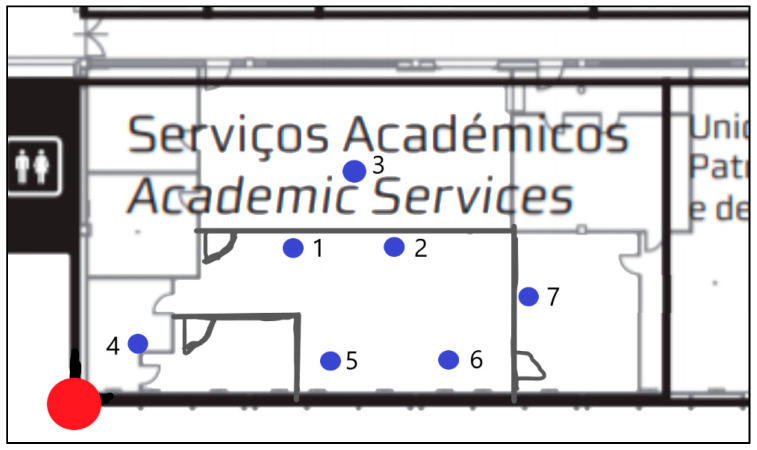
ISCTE-IUL University Academic Services, indoor sensors placement marked with blue circles and outdoor sensor marked with red circle.

Sensors were also placed at the Academic Services room, as shown in Figure 6. Indoor sensors are represented as a blue circle and the outdoor sensor as the red circle.

### 4.2. Network Layer

In this project, communication between the devices (nodes) and the application server was mediated by a LoRa server, implemented by CISCO at ISCTE-IUL, where all the sensors were added and configured, and the application was responsible for receiving and processing the data.

### 4.3. Application Layer

The application server is responsible for collecting the data from the network server and processing it. For this purpose, a Raspberry PI with a Raspbian operating system was configured to run a full-time web server using the Flash Web framework written in the Python programming language.

This server has the function of processing the incoming HTTP messages with the UPLINK data packets from the devices and creating the corresponding objects according to the sensor type (since the method of encoding the data varies according to the manufacturer/programming), sending them to a database located on the same Raspberry PI.

**Figure 7:**
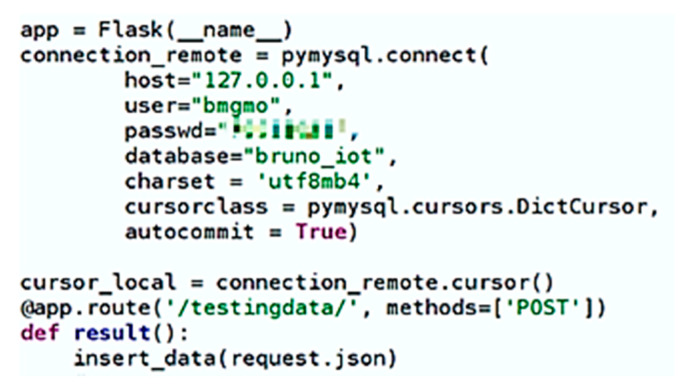
Flash Web Server developed in Python.

The configuration in Figure 7, due to the simplicity of the Flask framework, allows the creation of a server in a short time and with very little code, and the remaining lines correspond to the connection to the MySQL database, installed on Raspberry Pi.

**Figure 8:**
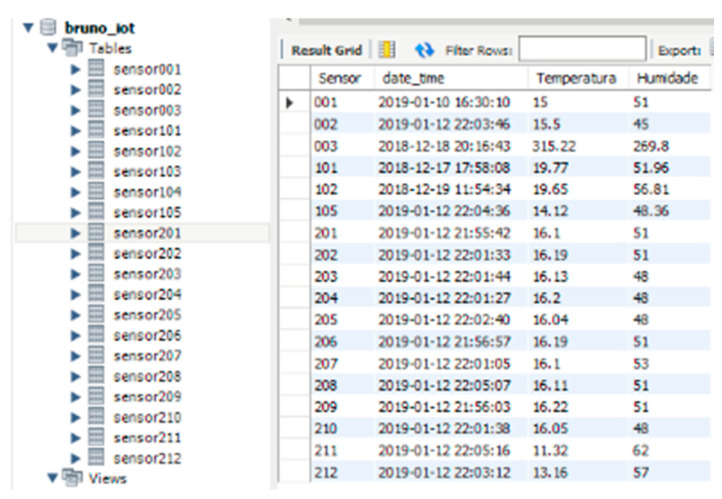
Query performed in the database, which indicates the last reading of each sensor.

The data collected by the sensors were structured into individual tables in a SQL database Figure 8 for analysis, consultation, and export. For Lansitec outdoor sensors, only temperature and humidity values were stored, while for the prototypes developed, other data were stored for further study of battery life, depending on code optimization and reading frequency as well as other indicators of the quality of the ISCTE LoRa network connection.

### 4.4. Viewing Dashboard

**Figure 9:**
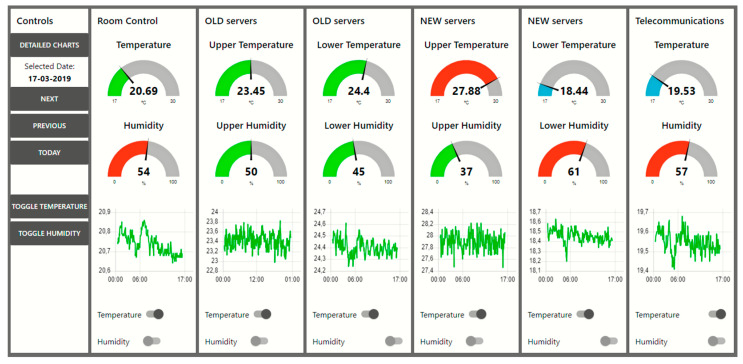
Dashboard main console displaying details about each sensor.

As an auxiliary display platform for reading each sensor, the open-source software Node-Red was used to display all the gathered information using charts and gauges Figure 9, which provide a graphical interface understandable by all types of users. This platform runs on the same Raspberry Pi as the database, which makes it easier to exchange information between them.

The dashboard allows the user to quickly detect anomalies on the temperature and humidity gauges that display real-time information as daily temperature charts that can be toggled to humidity. It also allows the user to visualize historical data by using the time controls on the left.

If a detailed analysis is required, the “detailed charts” tab displays all of the sensor data on the same charts. Each sensor can be toggled from the chart and the time tools are also available for historical visualization.

## 5. BIM Visualization Tool

By creating a 3D BIM-based visualization tool, we allowed users to observe and interact with the 3D model of the building through tablets placed at strategic places like corridors. This led to an increase in user interest, and real-time warnings provided the community with an overview of the current building state. This approach also decreased the time required for an alert to be dealt with and created awareness in the university community. The possibility of introducing gamification techniques into this approach could substantially increase its effectiveness.

A pre-existing 3D BIM of one of the university buildings was used and integrated with our system to evaluate this approach at the ISCTE-IUL University Campus, as described earlier. Based on a color-coding procedure, temperature values were coded according to comfort ranges. The winter comfort range was set between 21 °C and 32 °C and the summer comfort range was set between 27 °C and 37 °C [31,32]. When the temperature is below the lower limit, the color changes to shades of blue; if the temperature is between the comfort range, the color changes to shades of green; and if the temperature is above those values, the color changes to shades of red. A similar color-coding procedure applies to the humidity scales.

**Figure 10:**
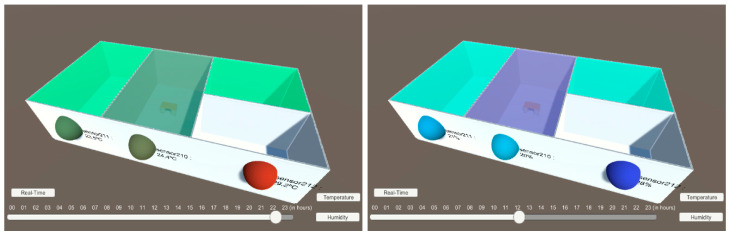
3D building imported from BIM with indoor sensors as volumes and outdoor sensors as spheres.

A simple 3D working model was first created to include individual objects, spheres for outdoor sensors, and volumes for indoor sensors Figure 10 to represent the sensor’s location and collected data.

**Figure 11:**
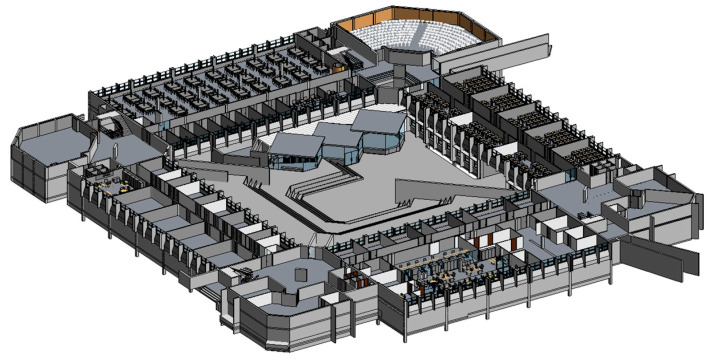
ISCTE-IUL Building I, 3D BIM developed with Autodesk’s Revit.

The 3D building was imported from the previously developed Autodesk’s Revit BIM presented in Figure 11.

A more detailed UI was created to allow historical data to be displayed using a calendar element and a time bar at the bottom of the screen. When time tools are not being used, data are updated in real-time. Aside from the color gradient, a label indicates the sensor’s name and variable value.

**Figure 12:**
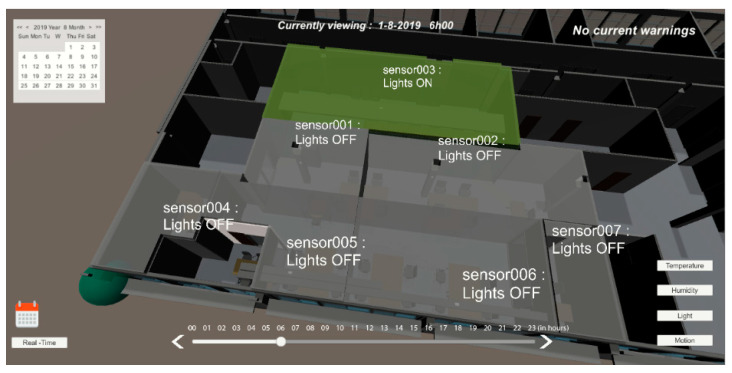
User interface with light mode selected for 01-08-2019 at 06:00 h.

On the main dashboard, users have several view modes for different variables such as temperature, humidity, motion, and light. Each of the views presents a warning signal when an event is detected, related to that variable. When the temperature mode is selected Figure 12, each monitored room presents a color related to the current temperature. If the current temperature is outside the comfort limits, the system displays a warning at the top right corner of the screen.

To prevent unnecessary energy waste when ACs are working, and people are opening windows without knowing if the outdoor temperature is worse than the indoor temperature, each sensor module has a red LED that lights up when the outdoor temperature is higher than the indoor temperature during summer, and the opposite during winter. If this situation still happens, by comparing the latest indoor temperature readings, it is possible to determine an anomaly when the AC is on, and the temperature keeps rising, triggering an alert to the person in charge. As sensor 007 room’s temperature was much lower than the outdoor temperature and adjacent rooms, the AC was determined as turned on, and because the motion sensor had a negative output for the past five minutes, the system displayed a warning.

**Figure 13:**
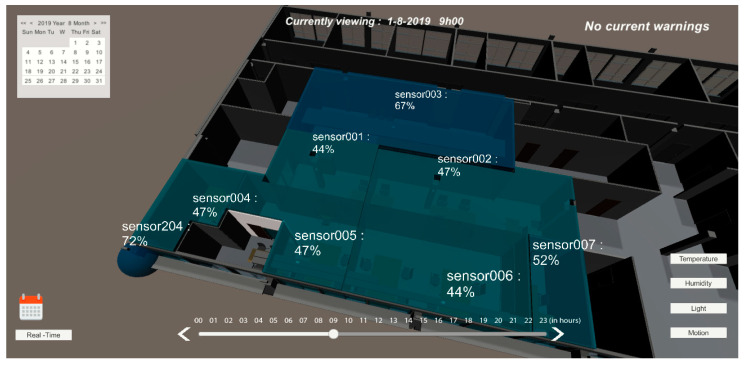
User interface with temperature mode selected for 01-08-2019 at 09:00 h.

In Figure 13, the user interface presents the relative humidity values for each sensor and creates a warning if any of the values are outside the comfort range.

**Figure 14:**
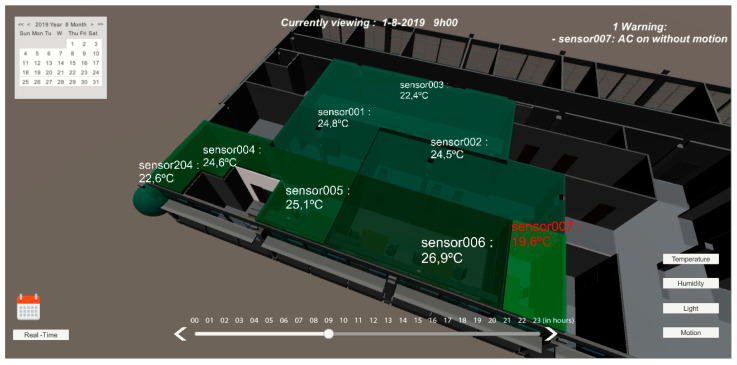
User interface with temperature mode selected for 01-08-2019 at 09:00 h.

Another dashboard view mode is light and motion, in which it is possible to observe the current light level determined by the light sensor and which rooms have artificial lights turned on Figure 14. When the natural light level is considered bright enough for work (based on a previous survey), the system detects that artificial light has been turned on and issues an alert to the 3D model warning about energy waste. In this view, users can identify warnings about rooms with artificial lights turned on and no motion detected. If the motion sensor of the room does not detect movement for more than five minutes and the light readings indicate that artificial lights are on, then a red warning is displayed on the 3D model, and it sends an email alert to the person in charge of that room.

## 6. Use Case Application at ISCTE

The IoT solution we developed holds enormous potential for environmental human behavior change with regard to energy usage in public and shared spaces. Individuals are more prone to engage in ecological actions when they have the right conditions, particularly in these settings. Interventions that create physical and social opportunities to protect the environment are the most successful [28]. Strategies such as providing eco-feedback are necessary to motivate individuals to be more aware of their environmental actions and their consequences. Furthermore, eco-feedback works best when individuals have access to real-time information that is context-specific [29]. Specifically, in the workplace, studies show that employees improve their environmental performance when they receive positive feedback on their initiative to lower energy consumption levels and know that their actions have a positive impact on their workplace settings [30]. Likewise, they are more willing to perform such actions when there is an opportunity to have an active role on the management of the resources, allowing them to exert their autonomy and control over the environmental conditions within these shared places (e.g., self-adjustable air-conditioning). On an organizational level, when the company has a positive attitude toward sustainability and supports pro-environmental practices, workers tend to feel more encouraged to build an environmentally friendly workspace [33]. This social stimulus is a helpful strategy given that employees share the same social space regularly and can easily observe each other’s performances, potentiating the creation of positive social shared norms and prompting cues for environmental protection at a group level [34].

Moreover, the use of color-coding techniques in the IoT system represents a smart and innovative way to convey environmental information to users. Color schemes allow information to be prioritized and highlighted according to the established goals and facilitate the model’s interpretation and legibility. These techniques have been shown to be very successful in increasing human awareness, attention, and information retention. Colored information tends to be more automatic and does not require as many mental resources to comprehend, resulting in more efficient information processing and decision making [37,38].

Through an enhanced and broader environmental overview, the IoT system works as an external monitoring resource that combines these features and allows users to self-monitor their ecological behavior and outcomes as well as to make more informed and conscious environmental decisions. As a result, individuals are more likely to feel encouraged to engage in a more active role and commit to their new environmentally-friendly behavior.

## 7. Results

Our implementation was performed at five controlled rooms based on the monitoring environment with sensors and related IoT described in the previous sections. From December 2019 to March 2020 (4-month data), we were able to detect more than 80 anomalies (30% related to artificial lights without any motion inside the rooms and the other 70% related with temperature warnings), considering that the system limits the temperature warnings to five per day. One of these five rooms was the secretary location, wherein an average of 20 people perform their work daily.

In these four months of testing, the 20 people would go to a web application (as an alternative to the mobile device) and select “Temperature too low” or a “Temperature too high”. Web applications in the first two months only allowed a user to give feedback about the room’s environment, and in the last two months, the sensor’s data were also displayed (internal room and external temperature). Data were represented in real-time dashboards as well as a 3D representation (BIM) presented above in Figure 12, Figure 13 and Figure 14; this representation was made with colors, taking into account the internationally defined comfort values. After the user expresses their ‘feeling’ at the temperature and light level, we received visual information about the other users’ thermal sensation in the same room, and we attributed an average color to all avatars in that room.

**Figure 15:**
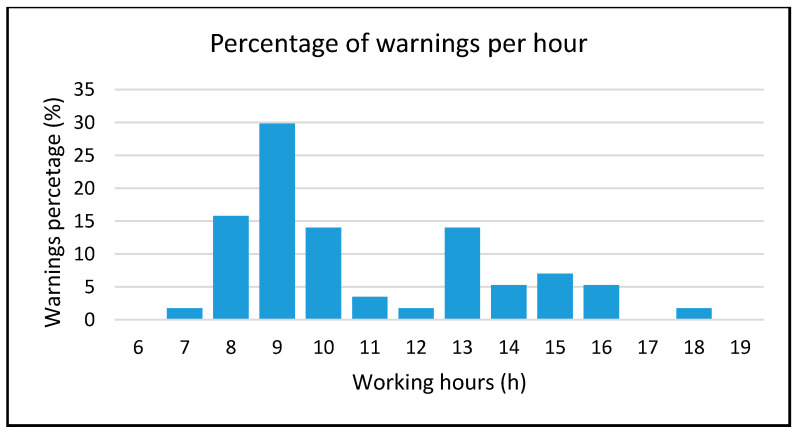
Percentage of warnings detected per hour.

In Figure 15, it is possible to identify the hours of the day when people feel less comfortable in their working places such as in the morning and after lunch. Morning hours (starting from 8 a.m. to 10 a.m.) are when people arrive and each room’s temperature is colder than it should be due to overnight temperature fluctuations.

**Figure 16:**
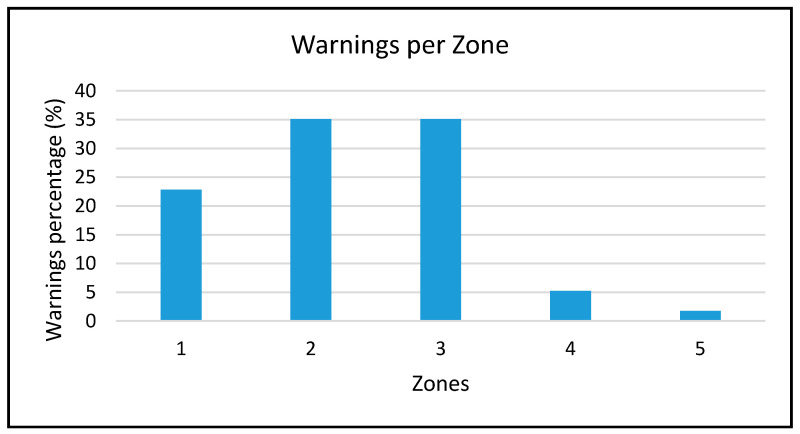
Percentage of warnings detected per zone.

Since each room or zone has its own solar exposure and number of windows, Figure 16 shows that we could detect that 92% of all warnings came from Zones 1, 2, and 3. These zones corresponded to areas with less solar exposure, which contributed to larger temperature gradients and a generally lower average temperature.

**Figure 17:**
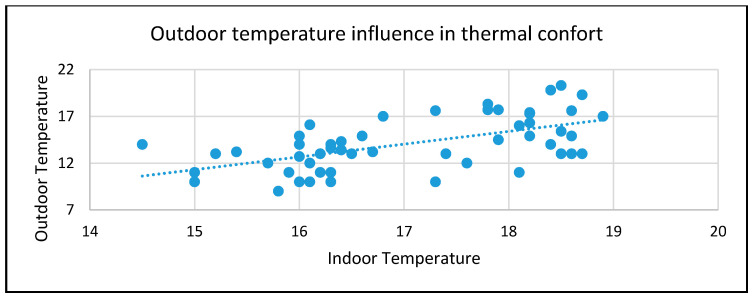
Relation between indoor thermal comfort and outdoor temperature, based on the indoor temperature when the warning was created.

With this study, we were able to identify a pattern relating to outdoor temperatures and thermal comfort. From Figure 17, we can identify the outdoor and indoor temperature when a certain warning was created. When the outdoor temperature was between 7 °C and 13 °C, there was a large cluster of warnings when the indoor temperature was less than 16.5 °C and this value can be an indicator of the lower thermal comfort limit. When the outdoor temperature increases to values between 13 °C and 21 °C, people indoors also require higher temperatures to feel comfortable. According to our data, this cluster of warnings can also indicate a lower thermal comfort limit for higher outdoor temperatures.

The tendency line in Figure 17 is very clear about the relation between thermal comfort and outdoor temperatures, which can be used by our system to determine the ideal temperature for each room to reduce energy consumption and maximize people’s comfort.

After analyzing the data, we were able to manage each room’s temperature according to their needs, since rooms with less solar exposure require more heating (in winter) than the others. The system can also reduce the ACs’ working time by establishing maximum temperatures for each room.

**Figure 18:**
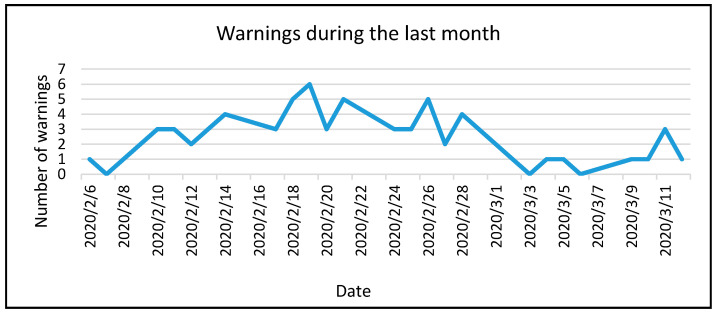
Number of warnings evolution during the last month.

During the final month, warnings were significantly reduced Figure 18 due to our system’s ability to give the user tools to have a real perception of the environment and take actions as an individual or as a group by understanding the overall room indicators.

The general opinion of a few members of the university community was positive and considered the system scalability to other rooms and buildings as a good solution to reduce temperature problems during winter and summer seasons.

In addition, facility managers responsible can visualize the collected data in a 3D scenario as created a better environment perception in a real-time process. 3D color information regarding temperature and energy consumption plays an important role, and we observed a consumption reduction of 4% to 5% in the monitored places. Furthermore, less temperature changes on heating/cooling systems were performed.

This approach can be extended to other applications such as water monitoring process.

## 8. Conclusions

In the current research work, a new approach to IoT integration was developed. Data collection, LoRa transmission, and visualization approaches were applied to understand data in their physical and functional environmental context. BIM allows data representation in a color-based visualization process, which greatly enhances people’s environmental perception in a *Things2People* interactive process. This visualization process is also used to give contextual information to both lay users and facility managers, who can see the distribution of values from a personalized perspective, and thus make appropriate adjustments. The research team is also working on an interactive process to be applied to energy management to measure and reduce energy consumption. 

With this research work, we were able to increase user interaction with the system since it was deployed using the BIM–Unity graphical interface, increase relative comfort in our test case rooms, drastically decrease of comfort temperature warnings by 75%, and finally, reduce energy consumption by presenting visual warnings to users of abnormal situations such as an empty room with the AC turned on.

**Figure 19:**
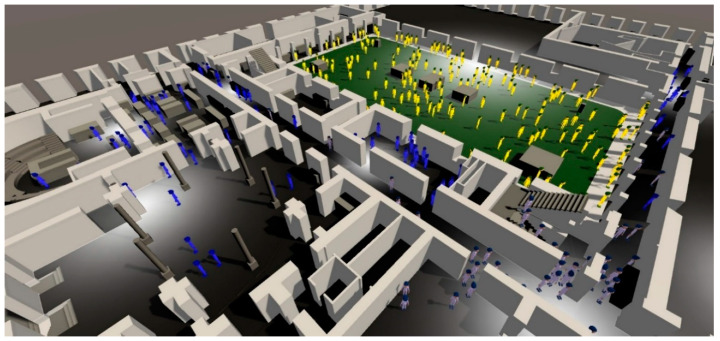
Users’ collective thermal perception. color-coded into avatars.

Through the visualization process, colored representation could be used to influence users and their thermal sensation can be used to create a collective thermal with the proposed avatar representation of Figure 19.

The majority of the developed methodology can be easily adapted to other cases, where previous building modeling information is available or developed. The rules that translate environment collected data into visual information can be pre-defined to achieve sustainability goals.

This research major contribution is the implementation of an IoT system with visual layers expressing different perspectives on top of a BIM-generated 3D visualization tool that can influence people’s perception of personal comfort, based on pre-defined configurable rules. Real-time interactions and information sharing are also important factors in user behavior modeling toward sustainability.

BIM visualization of people’s perception for temperature and light allows *People2People* interaction, where an individual feeling converges into a collective perception, and savings are accomplished by modeling selfish behaviors through this interactive process.

We propose a new approach to local user interaction using real environmental data and people sensation to change behavior toward savings actions. Mobile device interaction will play an important role. A tailored approach in information (incentives, norms,) provided the type of user (students, teachers) and usage (classroom, services) in an interaction. *Things2People* using 3D models also plays an important role.

Additional studies will be performed to measure the effects of *People2People* interaction using colored avatars and explore this in a focus group.
